# Effects of Bacterioruberin-Rich Haloarchaeal Carotenoid Extract on the Thermal and Oxidative Stabilities of Fish Oil

**DOI:** 10.3390/molecules28248023

**Published:** 2023-12-09

**Authors:** Fevziye Işıl Kesbiç, Hilal Metin, Francesco Fazio, Vincenzo Parrino, Osman Sabri Kesbiç

**Affiliations:** 1 Central Research Laboratory, Kastamonu University, 37150 Kastamonu, Turkey; ikesbic@kastamonu.edu.tr; 2 Institute of Science, Department of Sustainable Agriculture and Natural Sources, Kastamonu University, 37150 Kastamonu, Turkey; hilalmetin57@gmail.com; 3 Department of Veterinary Sciences, University of Messina, Viale Giovanni Palatucci, 13, 98168 Messina, Italy; 4 Department of Chemical, Biological, Pharmaceutical and Environmental Sciences, University of Messina, 98168 Messina, Italy; vincenzo.parrino@unime.it; 5 Faculty of Veterinary Medicine, Department of Animal Nutrition and Nutritional Diseases, Kastamonu University, 37150 Kastamonu, Turkey; okesbic@kastamonu.edu.tr

**Keywords:** *Halorubrum ezzemoulense*, natural antioxidants, TGA, thermal oxidation, fatty acid profile

## Abstract

This study aimed to assess the efficacy of a bacterioruberin-rich carotenoid extract (HAE) derived from the halophilic archaea *Halorubrum ezzemoulense* DSM 19316 in protecting crude fish oil against thermal oxidation. The research used fish oil derived from anchovies, which had a peroxide value (PV) of 6.44 ± 0.81 meq O_2_ kg^−1^. To assess the impact of HAE on the thermal stability and post-oxidation characteristics of fish oil, several concentrations of HAE were added to the fish oil samples: 0 ppm (no additive) (HAE0), 50 ppm (HAE50), 100 ppm (HAE100), 500 ppm (HAE500), and 1000 ppm (HAE1000). Furthermore, a control group was established with the addition of 100 ppm butylated hydroxytoluene (BHT100) in order to evaluate the effectiveness of HAE with a synthetic antioxidant that is commercially available. Prior to the fast oxidation experiment, thermogravimetric analysis was conducted on samples from all experimental groups. At the conclusion of the examination, it was seen that the HAE500 and HAE1000 groups exhibited a delay in the degradation temperature. The experimental groups underwent oxidation at a temperature of 55.0 ± 0.5 °C for a duration of 96 h. The measurement of PV was conducted every 24 h during this time. PV in all experimental groups exhibited a time-dependent rise (*p* < 0.05). However, the HAE500 group had the lowest PV measurement at the conclusion of the 96 h period (*p* < 0.05). Significant disparities were detected in the fatty acid compositions of the experimental groups at the completion of the oxidation experiment. The HAE500 group exhibited the highest levels of EPA, DHA, and ΣPUFA at the end of oxidation, with statistical significance (*p* < 0.05). Through the examination of volatile component analysis, specifically an oxidation marker, it was shown that the HAE500 group exhibited the lowest level of volatile components (*p* < 0.05). Consequently, it was concluded that the addition of HAE to fish oil provided superior protection compared to BHT at an equivalent rate. Moreover, the group that used 500 ppm HAE demonstrated the highest level of performance in the investigation.

## 1. Introduction

Aquaculture, farming aquatic organisms, has become an increasingly vital component of global food production in response to the rising demand for seafood products. As the world’s population grows and traditional fisheries face overexploitation and environmental challenges, aquaculture is a promising solution to ensure a sustainable and reliable supply of fish and other aquatic species for human consumption [1,2,3]. However, the sustainability of aquaculture operations is contingent upon various factors, with the composition of aquaculture feeds playing a crucial role [4,5]. Ironically, among the myriad ingredients used in these feeds, fish oil, derived primarily from wild-caught marine fish, has held a prominent position for its significant contributions to farmed aquatic species’ growth, health, and quality [6]. The future of aquaculture, which we have developed as an alternative to the natural resources used in feeding the world population, depends on the sustainability of natural resources and the efficient use of the resources we obtain from these resources [7].

Fish oil, rich in essential long-chain omega-3 fatty acids such as eicosapentaenoic acid (EPA) and docosahexaenoic acid (DHA), has been acknowledged for its multifaceted role in enhancing the nutritional value of aquaculture feeds [8]. These omega-3 fatty acids are indispensable components in the diets of many fish species due to their pivotal roles in growth, reproduction, immune function, and overall vitality [9,10,11]. Additionally, they have been associated with improving the end products’ taste, texture, and nutritional benefits, making them more appealing to fish [12]. Despite its undeniable importance, using fish oil in aquaculture feeds has raised concerns about its sustainability and environmental impact. The practice of relying on wild-caught fish to produce fish oil is not ecologically viable in the long term, given the strain it places on marine ecosystems and fish populations [13,14]. As a result, alternative sources of omega-3 fatty acids in aquaculture feeds have garnered increased attention in recent years. These alternatives include plant-based oils, algal oils, and genetically modified organisms; however, each has limitations such as fish health, growth performance and rentability of aquaculture practices [15,16,17,18,19]. For these reasons, using fish oil more efficiently is vital for extended-range fish farming.

The importance of antioxidants in preserving the quality and stability of fish oil within aquaculture feeds cannot be overstated [20]. Fish oil, rich in essential omega-3 fatty acids, is susceptible to oxidation when exposed to oxygen, light, and heat. This oxidative process can lead to the formation of harmful compounds, such as free radicals and peroxides [19], which not only compromise the nutritional value of the fish oil [21] but also give rise to off-flavors and rancidity [22,23]. Antioxidants play a pivotal role in safeguarding the integrity of fish oil by neutralizing these oxidative agents. By quenching free radicals and inhibiting the formation of peroxides, antioxidants help maintain the nutritional efficacy of fish oil while extending its shelf life [24,25]. This is of paramount significance in aquaculture, where the quality and health of farmed fish and aquatic species are closely tied to the nutritional composition of their feeds [26]. Consequently, incorporating antioxidants in fish oil protects the omega-3 fatty acids from oxidation, so they are retained until it is consumed, ultimately contributing to healthier and more sustainable aquaculture practices [27].

The use of antioxidants is very important for fish oil, which is extremely sensitive to oxidation. However, synthetic antioxidants are not always very reliable sources. Although some synthetic antioxidants were previously approved for use by the authorities, subsequent research has proven that they have toxic effects on organisms [28]. The most dramatic example of this situation is ethoxyquin [29,30]. For this reason, research and industrial applications in recent years have encouraged using natural antioxidants.

Carotenoids, a class of natural pigments widely found in various plants and microorganisms, have garnered significant attention in recent years for their remarkable role as antioxidants within aquaculture and beyond [31]. These compounds, notable for their colors ranging from red and orange to yellow and green, serve as potent antioxidants, safeguarding the health and quality of aquatic organisms [32]. Carotenoids, such as astaxanthin [33] and canthaxanthin [34], have been recognized for their ability to neutralize harmful free radicals and mitigate oxidative stress. In the context of aquaculture, the addition of carotenoids to fish feeds is a common practice to enhance the coloration of seafood, particularly in species like salmon and trout [35,36]. However, their significance extends beyond aesthetics. Carotenoids contribute to the overall health and immunity of aquatic species, acting as essential components in their diet [37]. Furthermore, these natural antioxidants play a pivotal role in protecting the fatty acids, including omega-3s, present in fish oil from oxidation, thereby ensuring that the nutritional value of aquaculture feeds remains intact [27]. As a result, the importance of carotenoids in aquaculture feeds transcends merely keeping fish healthy and aesthetically pleasing, also giving the fish oil in aquafeeds high protection from oxidation.

Bacterioruberin is a stable 50-carbon-bonded carotenoid previously thought to be found only in halophilic archaea [38]. However, recent research has shown it is present in other halophilic microorganisms, such as bacteria [39]. The conjugated double-bond system gives carotenoid molecules a robust and linear skeletal structure while providing a high reduction potential that makes these molecules potential antioxidants. The effect of carotenoids as antioxidants is assessed by their reaction with oxidizing agents and peroxide radicals. These properties play an important role in stabilizing carotenoids [40]. Bacterioruberin has 13 double conjugated bonds and thus functions as a hydroxyl free radical scavenger [41]. Since the number of double conjugated bonds it contains is higher than other natural carotenoids such as astaxanthin, it shows higher anti-oxidant activity than these carotenoids [42]. In addition to its effective antioxidant properties, bacterioruberin is a highly efficient source for industrial applications based on biosynthesis since it is obtained from halophilic organisms. The sanitation step is the most important cost and risk for bioactive molecules produced using microorganisms. When there is contamination in the process, the whole production is compromised, but the problems that may arise from sanitation are limited in processes using halophilic organisms. Halophilic organisms that thrive in high salinity media are more frequently preferred in industrial applications. In recent years, it has been proven by in vitro experiments that bacterioruberin shows a more qualified antioxidant effect than antioxidants such as butylated hydroxytoluene (BHT), which is frequently used in foods and fish oil [38]. Therefore, the main objective of this study was to elucidate the oxidative processes involved in the accelerated oxidation of feed-grade crude fish oil containing different concentrations of bacterioruberin-rich haloarchaeal extracts.

## 2. Results

### 2.1. Thermal Analysis

**Figure 1:**
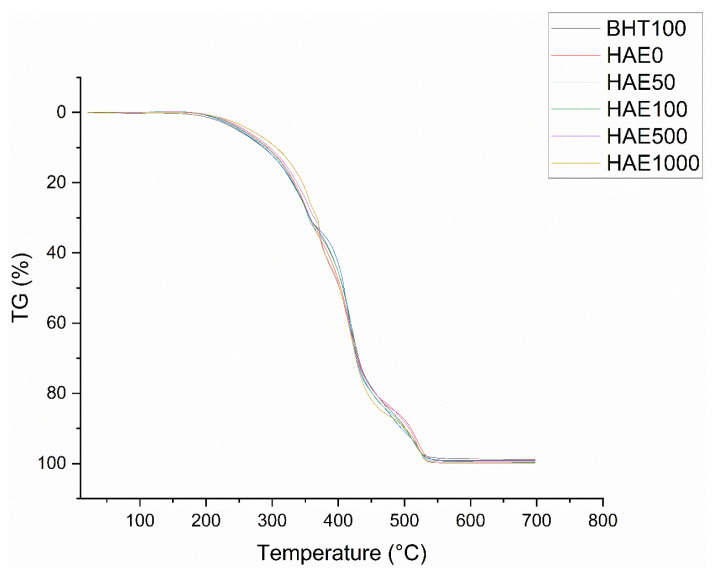
Investigating the impact of temperature on the weight loss of fish oils with varying ratios of HAE and 100 ppm BHT.

Thermogravimetric analysis (TGA) is a helpful method for investigating the alteration in the weight of samples as temperature rises. The decomposition temperature (Td) is the temperature at which the highest rate of weight loss occurs throughout each stage. The apex of the Td can be readily detected by analyzing the first derivative of the TGA curve about temperature, known as derivative thermogravimetry (DTG). According to Figure 1, the weight of fish oils with varying amounts of HAE dropped as the temperature rose from 25 to 700 °C. In addition to this, the recorded onset temperatures for Td, determined from the thermograms of the experimental groups, were as follows: BHT100 274.61 °C, HAE0 262.21 °C, HAE50 263.95 °C, HAE100 282.36 °C, HAE500 284.30 °C, and HAE1000 299.91 °C.

**Figure 2:**
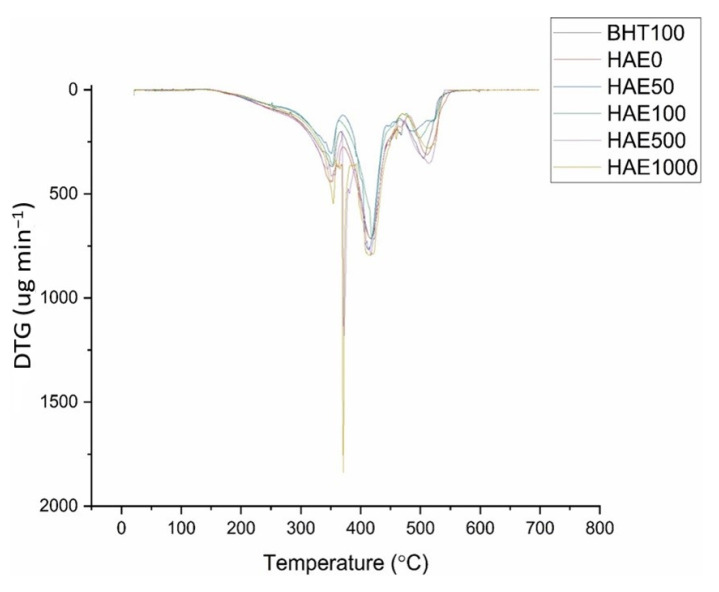
DTG curves of HAE and 100 ppm BHT in the atmosphere environment.

The temperature ranges for each stage of thermal decomposition, as determined from the relevant derivative curve (DTG) (Figure 2), are presented in Table 1.

### 2.2. Peroxide Value (PV)

**Figure 3:**
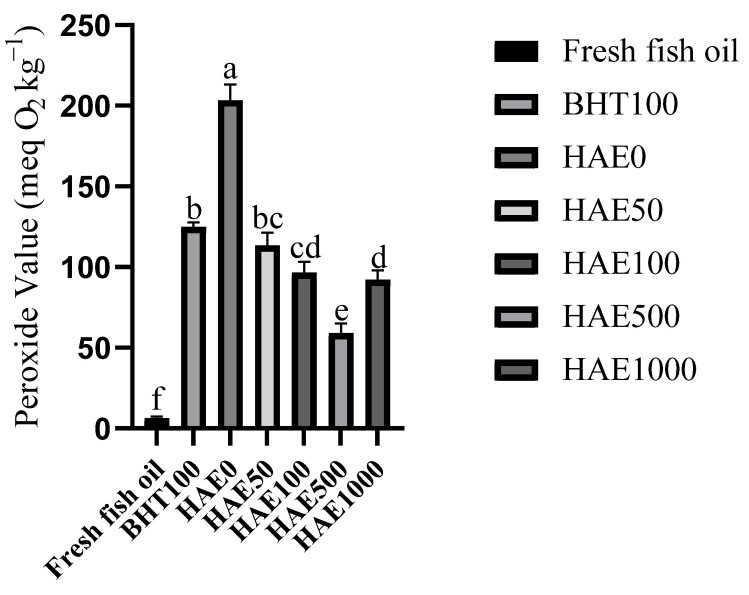
Effect on including HAE in various ratios and 100 ppm BHT to fish oils heated to 55 °C while continuously aerated on the development of peroxide after 96 h (*n* = 3). Significant differences between groups are indicated by values with different letters (*p* < 0.05).

The peroxide levels of fish oils with varying proportions of HAE were monitored every 24 h over a 96 h thermal oxidation experiment. The temporal fluctuation of PVs is displayed in Table 2. Furthermore, Figure 3 displays the peroxide values of the fish oil before and after the oxidation experiment.

### 2.3. Fatty Acid Profile

The alterations in the fatty acid compositions of the experimental fish oil groups, which were supplemented with HAE at various ratios, after the 96 h oxidation experiment, are displayed in Table 3.

Furthermore, variations were detected in the aromatic volatile constituents of fish oils due to oxidation. The variations in volatile components, which are classified as markers of rancid fish oil, among the different experimental groups are presented in Table 4.

## 3. Material and Methods

### 3.1. Haloarchaeal Strain and Culture Conditions

*Halorubrum ezzemoulense* DSM 19316 type strain (Genbank accession number: AM048786) was purchased from the Leibniz Institute DSMZ-German Collection of Microorganisms and Cell Cultures (DSMZ, Braunschweig, Germany). The medium composition used to cultivate *H. ezzemoulense* was carried out according to Kesbiç and Gültepe [39]. The cultures were incubated at 39 °C for seven days.

### 3.2. Preparation of Halophilic Archaeal Carotenoid Extracts

Carotenoid extraction was performed by the previously reported method. The culture was centrifuged at 9000 rpm for 20 min in the method. The supernatant was decanted and the pellet was washed briefly with phosphate buffer saline solution. After vortexing, the pellet was treated with 5 mL of methanol and sonicated for 60 min. After re-centrifugation, carotenoid was extracted. To calculate the amount of carotenoids, the carotenoid extract dissolved in methanol was transferred to a tared, sterile, light-proof glass bottle and all methanol was removed by nitrogen flow. When all the methanol had been removed, the bottle was weighed again, and the total carotenoid amount was calculated [38]. To determine the bacterioruberin content of the carotenoid extract, the methanolic extract was scanned in the spectrum between 300–600 nm and the characteristic bacterioruberin spectrum, with the two cis absorption maxima at around 369 and 387 and three peaks at 494, 527, and 467 nm with a broad shoulder, was observed in the UV-VIS Spectrophotometer (Epoch 2, BioTek Instruments, Inc., Winooski, VT, USA). Afterwards, as a result of the spectrophotometric measurement against methanol blank at 490 nm, the bacterioruberin content of the extract was calculated to be greater than 98% in the calculation made with the extinction coefficient method [43].

### 3.3. Oxidation Experiments Set-Up

The anchovy oil was purchased from a commercial factory (Kobyalar Group, Trabzon, Turkey) producing fish oil for aquafeed. The extracted bacterioruberin-rich haloarchaeal extract (HAE) was resolved in the anchovy oil. To investigate the protective impact of HAE on the thermal oxidation of fish oil, several experimental groups were formed by adding varying quantities of HAE to fish oil: 0 (HAE0), 50 (HAE50), 100 (HAE100), 500 (HAE500), and 1000 mg/kg (HAE1000). A 100 mg/kg butylated hydroxytoluene (BHT100) was used as the positive control [44]. Thermogravimetric analysis was performed on the groups containing different proportions of HAE. The experimental groups were placed in temperature-resistant bottles for 96 h while constantly illuminated and vented under thermal oxidation conditions at 55 ± 0.5 °C [19].

### 3.4. Thermal Decomposition of HAE-Doped Fish Oils by TGA

The decomposition of the HAE-doped fish oils were analyzed by a thermal gravimetric analyzer (TGA) (STA7300, Hitachi, Japan). An amount of 5–10 mg of the sample was placed in the TGA alumina pan and heated from 25 to 700 °C at 10 °C min^−1^ in an air atmosphere. The system collected data during the experiment to obtain the thermal decomposition rate curve [45].

### 3.5. Determination of Peroxide Values

The American Oil Chemists’ Society (AOCS) peroxide value determination method (Cd 8b-90) was used to measure the effect of thermal oxidation and the protective effect of HAE [46]. For this purpose, 0.5 g of samples from the control (fresh fish oil, not applied to the thermal oxidation procedure) and experimental groups were dissolved in 5 mL chloroform. The samples were stored in the dark at room temperature for 10 min after being treated with 15 mL of acetic acid and 1 mL of saturated potassium iodide. Following the waiting period, titration was performed in 75 mL of deionized water with 0.01 N sodium thiosulfate and a few drops of 1% starch as an indicator. Based on the titrant employed at the point of clear color development, signifying the conclusion of the titration, the peroxide value was determined using the following formula:

PV (%) = [(V1 − V0) N]/V

where V1 and V0 are the volumes of titrant used for the sample and blank, respectively, N is the normality of the titration solution, and M is the sample weight. The peroxide value assay findings were analyzed in triplicate as meq O_2_ kg^−1^ of oil.

### 3.6. Determination of Fatty Acid Compositions by GC-MS

The sample was prepared by combining 20 mg of the sample with 1 mL of 1 N sodium hydroxide in methanol, then heating the mixture at 80 °C for 15 min for saponification. The mixture was then heated at 110 °C for 15 min to facilitate transesterification before adding 1 mL of 14% boron trifluoride in methanol. Then, 1 mL of n-hexane was added, and the mixture was vortexed for 1 min before 3 mL of saturated sodium chloride solution was added. The resultant supernatant was used as the sample solution [47]. The fatty acid composition of oil samples was analyzed by the Shimadzu GC-MS QP 2010 ULTRA device (Shimadzu, Kyoto, Japan). The carrier gas was 99.99% purity helium and RTX-2330 capillary column (60 m; 0.25 mm; 0.20 μm) was used in the device. Column furnace temperature was 100 °C, injection temperature was 250 °C, interface temperature was 250 °C, ion source temperature was 200 °C, pressure was 90 kPa, and injection volume was 1 μL. Oven temperature program: 5 min at 100 °C, from 100 °C to 240 °C with 4 °C min^−1^ increase, and 15 min at 240 °C.

### 3.7. Determining the Volatile Compounds of Oxidized and Non-Oxidized Fish Oils by GC-MS

The secondary oxidation parameters were determined by analyzing the volatile compounds of the oil samples using GS-MS (Shimadzu GC-MS QP 2010 Ultra, Kyoto, Japan). The oil samples were treated with n-hexane and 1 μL of the extract was injected into the GC-MS equipped with a RXI-5MS capillary column (30 m × 0.25 mm i.d. × 0.25 μm). The carrier gas was 99.99% purity helium (1 mL/min), and the chromatographic conditions were as follows: the initial oven temperature was maintained at 50 °C for 5 min, then subsequently programmed from 50 to 270 °C at a rate of 5 °C/min, and it was held for another 5 min at 270 °C. Injector and interface temperatures were 250 °C, and the ion source temperature was 200 °C. Identification of the peaks was based on the comparison of their mass spectra with the spectra in the Wiley Data Library (Wiley W9N11) [39].

### 3.8. Statistical Analysis

The data were given as the mean and standard deviation (SD). The statistical data analysis was conducted using IBM SPSS Statistics version 20 software. One-way ANOVA and Tukey’s honestly significant difference (HSD) test were conducted to determine if there were any significant differences between the samples. The significance level used was *p* < 0.05.

## 4. Discussion

The use of chemical antioxidants, both in animal feed and human meals, has been a subject of controversy. Chemical antioxidants also delay oxidation and prolong the storage time of nutritious items. However, evidence suggests some antioxidants may have adverse health consequences on animals and humans [48,49]. In recent years, the safety and potentially harmful effects on human health of synthetic antioxidants have raised concerns, making them controversial [50]. For instance, in the European Union, the use of ethoxyquin was suspended under Regulation EC 2022/1375 and is now banned, as its use has been found to have adverse effects on human and animal health.

Halophilic archaea are highly salt-tolerant microorganisms, primarily classified in the family Haloferacaceae, belonging to the phylum Euryarchaeota in the domain Archaea [51]. The organisms are mostly aerobic and typically exhibit red pigmentation. These species are the primary microbial communities in highly saline conditions. Consequently, they necessitate a high level of salinity in the culture media. This requirement inhibits the contamination of other microorganisms in their culture media, so gives it an advantage in the utilization of halophilic archaea and their bioactive compounds in biotechnology. This restriction minimizes the risk of contamination during the cultivation process [52]. As previously mentioned, halophilic archaea produce pigment in shades of red; the major carotenoid is bacterioruberin [53]. Bacterioruberin and bacterioruberin-rich microbial extracts have high antioxidant activity. Therefore, such extracts have the potential to be used in many industrial products such as food, cosmetics, and pharmaceuticals. However, the potential of bacterioruberin in preserving items like fish oil, which are vulnerable to oxidation and may produce hazardous compounds when oxidized, has not been studied.

Thermogravimetric analysis (TGA) can measure the interaction between fish oil and oxygen by observing the weight loss of the fish oil as the temperature increases in the analysis output [45]. The current investigation yielded weight loss ranging from 260 to 540 °C. Previous research has indicated that variations in weight loss during thermal treatments can be mainly attributed to variances in the fatty acid composition of oils [54]. The current investigation used fish oil derived from anchovy. Nevertheless, prior research has indicated that oils derived from pink and red salmon experience varying degrees of weight reduction throughout distinct temperature intervals. The study demonstrated that the weight loss for red salmon oil occurred around 200–530 °C, while for pink salmon oil, it was approximately 200–610 °C [55]. The current study, together with earlier research, demonstrates that the origin of fish oil affects the temperature range in which weight loss occurs due to thermal treatment. The current investigation found that all fish oil supplementation postponed the onset temperature at which fish oil decomposes. The onset temperature of decomposition is a valuable instrument for comprehending the thermal endurance of the lipid supply. The greater the temperature at which an edible oil begins to break down, the greater its ability to withstand heat without deteriorating [56]. The study examined the temperature at which thermal degradation of fish oil made from anchovies begins to occur and found it to be 262.21 °C. Research on fish oils from various fish species has found that tuna oil has a degradation temperature of around 168 °C, whereas hoki oil has a degradation temperature of 172 °C [45]. It was observed that all HAE additions with a concentration higher than 50 ppm caused an increase in the first degradation temperature of fish oil, surpassing the positive control of 100 ppm BHT. The present study findings demonstrate that utilizing a microorganism-derived extract (HAE) is a superior method for enhancing the thermal stability of fish oil compared to employing an equivalent number of synthetic antioxidants (BHT). The study investigated the thermal degradation of fish oils, which happened across three distinct temperature ranges. This phenomenon is illustrated in Figure 2 as a combination of derivative curves. Previous thermal analysis studies on fish oil have reported comparable curves. Prior studies have indicated that the first, second, and third stages may correspond to the gradual breakdown of polyunsaturated fatty acids (PUFA), monounsaturated fatty acids (MUFA), and saturated fatty acids (SFA), followed by the formation of polymers and the evaporation of pyrolysis byproducts. Research has indicated that during phase 1, specifically the breakdown of polyunsaturated fatty acids (PUFAs) occurs [45]. By the onset of the degradation temperature of the first stage, the preservation of PUFAs is ensured. As has been established, these polyunsaturated fatty acids are the primary factor in fish oil’s importance. In this study, adding 500 and 1000 ppm HAE additives resulted in a postponement in the degradation temperature of the first stage, when compared to both the pure fish oil (HAE0) and the positive control group which had BHT added (BHT100).

Peroxide value (PV) is a crucial and extensively used parameter for measuring the extent of oxidation in oils, particularly fish oil. This method is used to ascertain the oxidation level in fish oil, often indicated by the presence of peroxide compounds, as a measure of rancidity. The PV value, commonly known as the primary oxidation indicator, is used to quantify the quality of oils [21,57]. Due to this rationale, several authorities use this characteristic to regulate the caliber of oils that include elevated levels of EPA and DHA, such as fish oil. According to experts, the PV values of oils, such as fish oil, should range from 5 to 10 meq O_2_ kg^−1^ based on their PV value [21]. The present investigation used fish oil with a peroxide value (PV) appropriate for the conditions. The peroxide value (PV) of the fish oil used in the research was determined to be 6.44 ± 0.81 milliequivalents of oxygen per kilogram (meq O_2_ kg^−1^). Several studies have revealed that the PV value, which serves as the main indicator of rancidity, rises in correlation with the duration of fish oil storage. Nevertheless, as shown in the present investigation, it is feasible to restrict the creation of PV by using antioxidants. In a previous investigation, the objective was to enhance the shelf life of fish oil derived from anchovies by using several tocopherol variants (α, γ, and δ). The research conducted at a temperature of 30 °C found that the non-additive fish oil attained a PV of 150 meq O_2_ kg^−1^ after 4 days, but the groups containing tocopherol did not reach this value even after 32 days [57]. An analogous phenomenon was identified in the present investigation. Analysis conducted at 24 h intervals throughout a 96 h heat treatment revealed a progressive rise in the PV value of the oil. Nevertheless, including HAE and BHT additions effectively shielded the fish oil from peroxide generation. Additionally, the pure fish oil’s PV value was greater than the other experimental groups in all measurements (*p* < 0.05). Another study was undertaken to safeguard fish oil against thermal oxidation using natural sources, further corroborating the present research results. In their study, Hasdemir et al. (2023) [19] demonstrated that the essential oil of *Borago officinalis*, which has been shown to possess antioxidant properties by in-vitro tests, effectively inhibited the generation of PV at the end of the thermal oxidation process of fish oil at a temperature of 70 °C for a duration of 24 h. Under some conditions, and often influenced by experimental conditions, carotenoids may lose their ability to scavenge radicals and instead exhibit a prooxidant nature. This phenomenon mostly happens at elevated oxygen pressure; however, it is also influenced by the carotenoid concentration [58]. β-carotene and lycopene behaved as prooxidants during the heat-catalyzed oxidation of safflower seed oil, specifically at quantities over 500 ppm [59]. In our investigation, we noticed a similar pattern, where the PV value significantly increased in groups with additives over 500 ppm like HAE1000. This is hypothesized to result from the prooxidant properties shown by the overuse of HAE.

Oxidation is the chemical process that occurs when unsaturated fatty acids present in fats and oils come into contact with oxygen. To clarify, oxidation in fats often refers to chemical reactions that alter the composition of polyunsaturated fatty acids. When PUFA-rich sources like fish oil undergo oxidation, there is a proportionate rise in SFA and MUFA groups, and a corresponding drop in PUFA group fatty acids [21]. An analogous pattern was discovered in the present investigation. After 96 h of temperature oxidation, there was a significant rise in the Σ SFA and Σ MUFA groups, whereas the Σ PUFA group had a substantial decline compared to fresh fish oil (*p* < 0.05). Comparable results were noted in a prior investigation to examine the composition of fish oil fatty acids throughout the deodorization process at various temperatures. As the temperature of deodorization in the research was raised, the ratios of Σ SFA and monounsaturated fatty acids Σ MUFA rose, but the ratios of polyunsaturated fatty acids Σ PUFA declined [60]. Nevertheless, HAE exhibited significantly superior efficacy compared to the positive control BHT in preserving fish oil’s fatty acid composition at the oxidation process’s end. Significantly, the oil from the 500 ppm (HAE500) group had the most similar fatty acid composition to fresh fish oil when compared to the other experimental groups after oxidation (*p* < 0.05). Eicosapentaenoic acid (EPA) and docosahexaenoic acid (DHA) are the primary fatty acids found in fish oil. These fatty acids belong to the polyunsaturated fatty acid (PUFA) group and are highly susceptible to oxidation. When fish oil becomes oxidized, EPA and DHA play a crucial role in degrading its quality. Hence, the primary objective of including antioxidants in fish oil is to inhibit the oxidation process of EPA and DHA. The present investigation demonstrated that the inclusion of HAE provided superior protection against EPA and DHA compared to the positive control BTH100. It was noted that the group receiving the 500 ppm supplementation showed higher protection of EPA and DHA compared to the other experimental groups, as shown by the PUFA data.

Various studies have revealed the production of certain aldehydes when fish oil containing EPA and DHA is subjected to heat. The present scenario permits the use of volatile flavor compounds as indicators of oil quality, providing insights into the deterioration of EPA and DHA during heat oxidation. Aldehydes have a key role in the taste of cooked fish/seafood since they have low threshold values and are formed when fatty acids and triglycerides break down via autoxidation, leading to the creation of hydroperoxides [61]. Research studies that test volatiles often use hexanal as a representative marker for lipid oxidation [62]. However, when discussing the oxidation of fish oil, it is insufficient to only refer to hexanal. Prior research has identified many volatile chemicals, notably hexanal, 2,4-heptadienal, and 2,6-nonadienal, after the oxidation of fish oil [21,63,64]. Similar findings were found in the current study. After 96 h of heat oxidation, it was found that the oil without addition (HAE0) had the greatest concentration of volatile chemicals, whereas fresh fish oil had essentially no detectable volatile compounds. Additionally, it was noted that the generation of volatile compounds was inhibited based on the rate of HAE additive. The HAE500 group exhibited a volatile compound profile resembling fresh oil (*p* < 0.05).

## 5. Conclusions

Consequently, it was concluded in the current thermally induced accelerated rancidity study that including a bacterioruberin-rich extract obtained from halophilic archaea in fish oil safeguards the stability of the fish oil and preserves its biochemical composition from thermal oxidation at 55 °C during a period of 96 h. In the present investigation, it has been shown that BHT, a synthetic substance used to safeguard the quality of food and feed items from oxidation, effectively preserves fish oil. However, a current study has shown that using haloarcheal extract at an equivalent dosage provides superior protection by suppressing PV and oxidation-induced volatile molecule formation in fish oil compared to the use of BHT. Expressly, it was noted that the use of 500 ppm (HAE) offers the most effective safeguarding of fish oil with regards to its bio-physico-chemical preservation. 

In future studies, it is recommended to investigate alternative natural sources that can protect fish oil against oxidation and to test the performance of bacterioruberin-rich haloarchaeal carotenoid extracts in long-term storage of fish oil.

## Figures and Tables

**Table 1:** Temperatures of thermal decomposition stages of varying ratios of HAE and 100 ppm BHT.

	Stage 1 (°C)	Stage 2 (°C)	Stage 3 (°C)
BHT100	357.91	417.24	505.73
HAE0	352.13	418.18	514.64
HAE50	349.3	413.66	513.15
HAE100	351.28	418.28	498.29
HAE500	383.77	417.63	514.77
HAE1000	370.61	420.68	512.43

**Table 2:** Time-dependent PV values of anchovy oils containing different ratios of haloarchaeal extract and 100 ppm BHT.

Experimental Groups	BHT100	HAE0	HAE50	HAE100	HAE500	HAE1000	*p*-Value
Hours							
24	15.57 (5.15) ^d/ab^	22.44 (1.72) ^d/a^	16.46 (3.04) ^c/ab^	14.00 (2.29) ^c/ab^	17.23 (2.44) ^b/ab^	11.37 (1.48) ^c/b^	0.013
48	32.22 (3.36) ^c/b^	60.76 (5.82) ^c/a^	27.15 (2.29) ^c/b^	23.61 (2.01) ^c/bc^	16.27 (2.93) ^b/c^	17.53 (3.14) ^c/c^	<0.001
72	27.60 (4.92) ^b/b^	107.38 (7.77) ^b/a^	87.47 (9.43) ^b/b^	79.27 (2.05) ^b/b^	25.73 (1.76) ^b/c^	75.80 (4.58) ^b/b^	<0.001
96	124.98 (2.60) ^a/b^	203.41 (9.90) ^a/a^	113.51 (7.76) ^a/bc^	96.64 (6.50) ^a/cd^	58.97 (6.09) ^a/e^	92.09 (5.86) ^a/d^	<0.001
*p*-value	<0.001	<0.001	<0.001	<0.001	<0.001	<0.001	

The data are shown as the mean (SD) (*n* = 3). The first superscript indicates the variation in time on the PV value (column), whereas the second superscript indicates the variation in experimental groups (row) (*p* < 0.05).

**Table 3:** Fatty acid profile of haloarchaeal extract and 100 ppm BHT after 96 h oxidation experiment (g 100 g^−1^).

Fatty Acid	Fresh Fish Oil	BHT100	HAE0	HAE50	HAE100	HAE500	HAE1000	*p*-Value
C14:0	5.38 (0.29) ^c^	6.23 (0.04) ^ab^	6.69 (0.03) ^a^	6.19 (0.05) ^ab^	5.70 (0.56) ^bc^	5.88 (0.05) ^bc^	5.94 (0.03) ^bc^	<0.001
C15:0	1.28 (0.03) ^d^	1.45 (0.02) ^b^	1.59 (0.01) ^a^	1.46 (0.01) ^b^	1.43 (0.01) ^bc^	1.38 (0.01) ^c^	1.42 (0.01) ^bc^	<0.001
C16:0	16.81 (0.70) ^e^	20.22 (0.04) ^b^	21.66 (0.15) ^a^	19.88 (0.06) ^bc^	19.21 (0.17) ^cd^	19.22 (0.05) ^cd^	18.86 (0.03) ^d^	<0.001
C17:0	1.30 (0.02) ^c^	1.43 (0.02) ^b^	1.54 (0.02) ^a^	1.42 (0.01) ^b^	1.36 (0.04) ^bc^	1.34 (0.017) ^c^	1.35 (0.028) ^c^	<0.001
C18:0	4.94 (0.17) ^e^	5.90 (0.26) ^b^	6.31 (0.04) ^a^	5.81 (0.04) ^bc^	5.66 (0.06) ^cd^	5.50 (0.035) ^d^	5.52 (0.01) ^d^	<0.001
C20:0	0.72 (0.02) ^e^	0.81 (0.01) ^bc^	0.90 (0.01) ^a^	0.84 (0.01) ^b^	0.80 (0.01) ^c^	0.76 (0.01) ^d^	0.79 (0.01) ^cd^	<0.001
∑SFA	30.43 (0.70) ^d^	36.04 (0.12) ^b^	38.71 (0.19) ^a^	35.62 (0.13) ^b^	34.17 (0.82) ^c^	34.10 (0.08) ^c^	33.89 (0.05) ^c^	<0.001
C16:1n-7	8.5 (0.3) ^b^	9.57 (0.05) ^a^	9.78 (0.38) ^a^	9.59 (0.08) ^a^	9.42 (0.01) ^a^	9.36 (0.06) ^a^	9.32 (0.03) ^a^	<0.001
C16:1n-10	0.94 (0.1) ^a^	0.88 (0.01) ^a^	0.86 (0.06) ^a^	0.89 (0.01) ^a^	0.87 (0.01) ^a^	0.84 (0.01) ^a^	0.9 (0.02) ^a^	0.271
C18:1n-9	12.05 (0.34) ^d^	13.54 (0.04) ^b^	14.12 (0.04) ^a^	13.32 (0.11) ^bc^	13.21 (0.03) ^bc^	13.05 (0.06) ^c^	13.21 (0.02) ^bc^	<0.001
C18:1n-7	2.52 (0.12) ^c^	2.96 (0.04) ^b^	3.14 (0.03) ^a^	2.95 (0.03) ^b^	2.91 (0.015) ^b^	2.82 (0.01) ^b^	2.89 (0.01) ^b^	<0.001
C20:1n-9	1.76 (0.10) ^b^	1.98 (0.06) ^ab^	2.02 (0.03) ^ab^	2.24 (0.23) ^a^	1.98 (0.11) ^ab^	1.94 (0.2) ^ab^	2.08 (0.10) ^ab^	0.030
C22:1n-9	1.13 (0.12) ^b^	1.11 (0.01) ^b^	1.17 (0.01) ^ab^	1.12 (0.02) ^b^	1.11 (0.01) ^b^	1.07 (0.01) ^b^	1.77 (0.58) ^a^	0.022
∑MUFA	26.92 (0.28) ^d^	30.06 (0.07) ^abc^	31.11 (0.48) ^a^	30.12 (0.36) ^abc^	29.53 (0.11) ^bc^	29.09 (0.30) ^c^	30.18 (0.7) ^ab^	<0.001
C18:2n-6	1.94 (0.05) ^a^	2.03 (0.02) ^a^	1.98 (0.05) ^a^	1.99 (0.005) ^a^	2.03 (0.05) ^a^	1.96 (0.01) ^a^	2.02 (0.01) ^a^	0.092
C18:3n-6	0.22 (0.01) ^b^	0.21 (0.01) ^b^	0.24 (0.01) ^a^	0.22 (0.01) ^ab^	0.22 (0.01) ^ab^	0.22 (0.01) ^ab^	0.22 (0.01) ^b^	0.021
C18:3n-3 (ALA)	2.18 (0.07) ^a^	2.09 (0.03) ^ab^	1.83 (0.11) ^c^	2.03 (0.02) ^b^	2.10 (0.01) ^ab^	2.17 (0.02) ^ab^	2.15 (0.02) ^ab^	<0.001
C20:4n-6	1.01 (0.03) ^a^	0.82 (0.01) ^d^	0.72 (0.04) ^e^	0.84 (0.01) ^cd^	0.89 (0.01) ^bc^	0.88 (0.02) ^bcd^	0.93 (0.02) ^b^	<0.001
C20:3n-3	0.69 (0.06) ^a^	0.5 (0.01) ^cd^	0.45 (0.04) ^d^	0.58 (0.01) ^bc^	0.60 (0.01) ^ab^	0.55 (0.02) ^bc^	0.61 (0.017) ^ab^	<0.001
C20:5n-3 (EPA)	9.53 (0.15) ^a^	7.76 (0.04) ^d^	6.47 (0.07) ^e^	7.79 (0.03) ^d^	8.34 (0.02) ^c^	8.55 (0.13) ^bc^	8.59 (0.04) ^b^	<0.001
C22:6n-3 (DHA)	18.53 (0.11) ^a^	14.26 (0.21) ^e^	11.05 (0.03) ^f^	14.0 (0.26) ^e^	14.93 (0.06) ^d^	16.15 (0.13) ^b^	15.67 (0.12) ^c^	<0.001
C22:5n-6	0.82 (0.01) ^a^	0.61 (0.01) ^d^	0.50 (0.01) ^e^	0.61 (0.01) ^d^	0.66 (0.01) ^c^	0.7 (0.01) ^b^	0.68 (0.01) ^bc^	<0.001
ΣPUFA	34.95 (0.22) ^a^	28.3 (0.18) ^d^	23.27 (0.27) ^e^	28.08 (0.25) ^d^	29.81 (0.08) ^c^	31.21 (0.11) ^b^	30.89 (0.13) ^b^	<0.001
Others	7.68 (1.03) ^a^	5.59 (0.25) ^bc^	6.89 (0.54) ^ab^	6.17 (0.73) ^abc^	6.48 (0.64) ^abc^	5.59 (0.14) ^bc^	5.03 (0.79) ^c^	0.004

The data are shown as the mean (SD) (*n* = 3). The superscripts show significant difference (*p* < 0.05).

**Table 4:** Mean headspace concentration of volatile organic compounds present in fish oil rancidized by thermal treatment.

Volatile Molecule (%)	Fresh Fish Oil	BHT100	HAE0	HAE50	HAE100	HAE500	HAE1000	*p*-Value
Hexenal	0.00 (0.00) ^e^	1.19 (0.07) ^bc^	1.56 (0.15) ^a^	1.26 (0.02) ^b^	1.06 (0.02) ^bc^	0.77 (0.03) ^d^	1.01 (0.07) ^c^	<0.001
2,4-Heptadienal	0.00 (0.00) ^e^	1.06 (0.15) ^bc^	1.35 (0.06) ^a^	1.21 (0.01) ^ab^	0.99 (0.03) ^c^	0.37 (0.04) ^d^	0.90 (0.03) ^c^	<0.001
2,4-Decadienal	0.00 (0.00) ^e^	0.71 (0.05) ^b^	0.94 (0.04) ^a^	0.71 (0.04) ^b^	0.60 (0.07) ^bc^	0.40 (0.01) ^d^	0.52 (0.04) ^cd^	<0.001
Hexadecane	0.54 (0.19) ^d^	1.57 (0.35) ^c^	2.58 (0.31) ^a^	2.23 (0.09) ^ab^	1.78 (0.16) ^cb^	0.97 (0.05) ^d^	1.96 (0.04) ^cb^	<0.001
2,6-Nonadienal	0.00 (0.00) ^f^	1.94 (0.17) ^b^	2.58 (0.20) ^a^	1.82 (0.10) ^bc^	1.43 (0.06) ^de^	1.22 (0.03) ^e^	1.57 (0.03) ^cd^	<0.001

The data are shown as the mean (SD) (*n* = 3). The superscripts show a significant difference (*p* < 0.05).

## Data Availability

Data supporting the conclusions of this manuscript are included within the article.
